# How to reveal people’s preferences: Comparing time consistency and predictive power of multiple price list risk elicitation methods

**DOI:** 10.1007/s11166-016-9247-6

**Published:** 2017-02-01

**Authors:** Tamás Csermely, Alexander Rabas

**Affiliations:** 1 University of Vienna, Doctoral School of Operations Management and Logistics, Oskar Morgenstern Platz 1, 1090 Vienna, Austria; 2 Vienna University of Economics and Business, Institute for Public Sector Economics, Vienna, Austria; 3 Lauder Business School, Vienna, Austria

**Keywords:** Risk, Multiple price list, MPL, Revealed preferences, Risk preference elicitation methods, C91, D81

## Abstract

The question of how to measure and classify people’s risk preferences is of substantial importance in the field of economics. Inspired by the multitude of ways used to elicit risk preferences, we conduct a holistic investigation of the most prevalent method, the multiple price list (MPL) and its derivations. In our experiment, we find that revealed preferences differ under various versions of MPLs as well as yield unstable results within a 30-minute time frame. We determine the most stable elicitation method with the highest forecast accuracy by using multiple measures of within-method consistency and by using behavior in two economically relevant games as benchmarks. A derivation of the well-known method by Holt and Laury (American Economic Review **92**(5):1644–1655, 2002), where the highest payoff is varied instead of probabilities, emerges as the best MPL method in both dimensions. As we pinpoint each MPL characteristic’s effect on the revealed preference and its consistency, our results have implications for preference elicitation procedures in general.

**Electronic supplementary material:**

The online version of this article (doi:10.1007/s11166-016-9247-6) contains supplementary material, which is available to authorized users.

## Introduction

Risk is a fundamental concept that affects human behavior and decisions in many real-life situations. Whether a person wants to invest in the stock market, tries to select the best health insurance or just wants to cross the street, he/she will face risky decisions every day. Therefore, risk attitudes are of high importance for decisions in many economics-related contexts. A multitude of studies elicit risk preferences in order to control for risk attitudes, as it is clear that they might play a relevant role in explaining results — e.g. De Véricourt et al. (2013) in the newsvendor setting, Murnighan et al. (1988) in bargaining, Beck (1994) in redistribution or Tanaka et al. (2010) in linking experimental data to household income, to name just a few. Moreover, several papers try to shed light on the causes of risk-seeking and risk-averse behavior in the general population with laboratory (Harrison and Rutström 2008), internet (Von Gaudecker et al. 2011) and field experiments (Andersson et al. 2016; Harrison et al. 2007). Since the seminal papers by Holt and Laury (2002, 2005), approximately 20 methods have been published which provide alternatives to elicit risk preferences. They differ from each other in terms of the varied parameters, representation and framing. Many of these risk elicitation methods have the same theoretical foundation and therefore claim to measure the same parameter — a subject’s “true” risk preference. However, there are significant differences in results depending on the method used, as an increasing amount of evidence suggests. It follows that if someone’s revealed preference is dependent on the measurement method used, scientific results and real-world conclusions might be biased and misleading.

As far as existing comparison studies are concerned, they usually compare two methods with each other and often use different stakes, parameters, framing, representation, etc., which makes their results hardly comparable. Our paper complements existing experimental literature by making the following contribution: Taking the method by Holt and Laury (2002) as a basis, we conduct a comprehensive comparison of the multiple price list (MPL) versions of risk elicitation methods by classifying all methods into nine categories. To the best of our knowledge, no investigation — including various measures of between- and within-method consistency — has ever been conducted in the literature that incorporates such a high number of methods. To isolate the effect of different methods, we consistently use the MPL representation and calibrate the risk intervals to be the same for each method assuming expected utility theory (EUT) and constant relative risk aversion (CRRA), while also keeping the risk-neutral expected payoff of each method constant and employing a within-subject design. Moreover, our design allows us to investigate whether differences across methods can be reconciled by assuming different functional forms documented in the literature such as constant absolute risk aversion (CARA), decreasing relative risk aversion (DRRA), decreasing absolute risk aversion (DARA), increasing relative risk aversion (IRRA) and increasing absolute risk aversion (IARA). Additionally, we extend our analysis to incorporate EUT with probability weighting and also to incorporate prospect theory (PT) and cumulative prospect theory (CPT).

We investigate the within-method consistency of each method by comparing the differences in subjects’ initial and repeated decisions within the same MPL method. Moreover, we assess methods’ self-perceived complexity and shed light on differences and similarities between methods. In the end, we provide suggestions for which specific MPL representation to use by comparing our results to decisions in two benchmark games that resemble real-life settings: investments in capital markets and auctions. Therefore, we analyze the methods along two dimensions, robustness and predictive power, and determine which properties of particular methods drive risk attitude and its consistency.

We find that a particular modification of the method by Holt and Laury (2002) derived by Drichoutis and Lusk (2012, 2016) has the highest predictive power in investment settings both according to the OLS regression and Spearman rank correlation. In addition, specific methods devised by Bruner (2009) also perform relatively well in these analyses. However, the method by Drichoutis and Lusk (2012, 2016) clearly outperforms the other methods in terms of within-method consistency and is perceived as relatively simple — in the end, our study provides the recommendation for researchers to implement this method when measuring risk attitudes using an MPL framework. Moreover, our results remain qualitatively the same if we relax our assumption on the risk aversion function, or if we take probability weighting or alternative theories such as prospect theory or cumulative prospect theory into account.

### Multiple price lists explained

Incentivized risk preference elicitation methods aim to quantify subjects’ risk perceptions based on their revealed preferences. We present nine methods in a unified structure — the commonly used MPL format — to our subjects, taking one of the most cited methods as a basis: Holt and Laury (2002). The MPL table structure is as follows: Each table has multiple rows, and in each row all subjects face a lottery (two columns) on one side of the table, and a lottery or a certain payoff (one or two columns) on the other side, depending on the particular method. Then, from row to row, one or more of the parameters change. The methods differ from each other by the parameter which is changing. As the options on the right side become strictly more attractive from row to row, a subject indicates the row where he/she wants to switch from the left option to the right option. This switching point then gives us an interval for a subject’s risk preference parameter according to Table 1,^1^ assuming EUT and CRRA^2^.

Note that several other representations of risk elicitation methods exist besides the MPL such as the bisection method (Andersen et al. 2006), the trade-off method (Wakker and Deneffe 1996), questionnaire-based methods (Weber et al. 2002), willingness-to-pay (Hey et al. 2009), etc., but the MPL is favored because of its common usage. Andersen et al. (2006) consider that the main advantage of the MPL format is that it is transparent to subjects and it provides simple incentives for truthful preference revelation. They additionally list its simplicity and the little time it takes as further benefits. As far as the specific risk elicitation method in the MPL framework designed by Holt and Laury (2002) is concerned, it has proven itself numerous times in providing explanations for several phenomena such as behavior in 2x2 games (Goeree et al. 2003), market settings (Fellner and Maciejovsky 2007), smoking, heavy drinking, being overweight or obese (Anderson and Mellor 2008), consumption practices (Lusk and Coble 2005) and many others.

Early studies document violations of EUT under risky decision making and provide suggestions how these differences can be reconciled (Bleichrodt et al. 2001). In addition, recent studies (Tanaka et al. 2010; Bocqueho et al. 2014) document potential empirical support for prospect theory (PT, Kahneman and Tversky (1979))^3^ when it comes to risk attitudes: Harrison et al. (2010) found that PT describes behavior of half of their sample best. There is also evidence that subjective probability weighting (PW) (Quiggin 1982) should be taken into account. In addition, cumulative prospect theory (CPT) — PT combined with PW (Tversky and Kahneman 1992) — might also be a candidate that can explain the documented anomalies under EUT. Wakker (2010) provides an extensive review on risk under PT.

We justify using CRRA as Wakker (2008) claims that it is the most commonly postulated assumption among economists. Most recently, Chiappori and Paiella (2011) provide evidence on the validity of this assumption in economic-financial decisions.^4^ Nevertheless, alternative functional forms have been introduced, e.g. CARA^5^ (Pratt 1964). It was also questioned whether social status — and mostly the role of wealth or income — might shape risk attitude, which would lead to functions which are increasing or decreasing in these factors such as IRRA and DRRA (Andersen et al. 2012)^6^ or IARA and DARA (Saha 1993).^7^ A review of these functions is provided by Levy (1994). In our robustness analysis, we relax our original assumptions on EUT and CRRA and incorporate all of the above mentioned alternative theories and functional forms. Note that even though we calibrated our parameters to accomodate EUT and CRRA, one is still able to calculate the risk parameter *ρ* using the aforementioned alternative specifications.^8^

We group our aforementioned nine risk elicitation methods into two categories:

1. The standard gamble methods (SG methods), where on one side of the table there is always a 100% chance of getting a particular certain payoff and on the other side there is a lottery.
2. The paired gamble methods (PG methods), with lotteries on both sides.

We therefore primarily conduct a comparison of different MPL risk elicitation methods. What we do not claim, however, is that the method devised by Holt and Laury (2002) (or MPL in general) is the most fitting to measure people’s risk preferences — we strive to give a recommendation to researchers who already intend to use Holt and Laury (2002) in their studies, and provide a better alternative that shares its attributes with the original MPL design.

It should be mentioned that there is an alternative interpretation of our study: The different MPL methods can also be conceived as a mapping of existing risk elicitation methods (from other frameworks) to the MPL space. Several methods exist where the risk elicitation task is provided in a framed context — such as pumping a balloon until it blows (Lejuez et al. 2002) or avoiding a bomb explosion (Crosetto and Filippin 2013). Similarly, some methods differ due to the representation of probabilities with percentages (Holt and Laury 2002) or charts (Andreoni and Harbaugh 2010). All these methods can be displayed with different MPLs by showing the probabilities and the corresponding payoffs in a table format. We provide a complete classification of these methods in the Literature Review section.

Up to now, different risk elicitation methods were compared by keeping the original designs, but this approach comes at a price: As the methods differ in many dimensions, any differences found can be attributed to any of those particular characteristics. Our approach can be understood as a way to make all risk elicitation methods as similar as possible, with the drawback of losing the direct connection to the original representation. This paper should therefore primarily be seen as a comparison of different MPL risk elicitation methods, and the resulting comparison of existing risk elicitation methods by mapping them into the same space is only reported for the sake of completeness.

### Literature review

We will now discuss the different methods in greater detail and how they are embedded in the literature, if at all. Table 2 provides a summary of the exact parameter that is changing across methods.^9^

#### Standard gamble methods

Among the SG methods, there are four parameters that can be changed: The sure payoff (*sure*), the high payoff of the lottery (*high*), the low payoff of the lottery (*low*) or the probability of getting the high payoff (*p*) (or the probability of getting the low payoff (1−*p*), respectively). The parameters must of course be chosen in such a way that *h* *i* *g* *h*>*s* *u* *r* *e*>*l* *o* *w* always holds. For instance, we denote the SG method where the low payoff is changing by “SGlow”, the SG method with the varying certainty equivalent by “SGsure” or the standard gamble method where the probabilities are changing as “SGp”.

Binswanger (1980) introduced a method (SGall) where only one of the options has a certainty equivalent. The other options consist of lotteries where the probabilities are fixed at 50-50, but both the high and the low payoff are changing. Cohen et al. (1987) used risk elicitation tasks in which probabilities and lottery outcomes were held constant and only the certainty equivalent was varied. These methods have later been redesigned and presented in a more sophisticated format as a single choice task by Eckel and Grossman (2002, 2008).

A recent investigation by Abdellaoui et al. (2011) presents a similar method (SGsure method) in an MPL format with 50-50 probabilities. Bruner (2009) presents a particular certainty equivalent method, where the certainty equivalent and the lottery outcomes are held constant, but the corresponding probabilities of the lotteries are changing (SGp method). Additionally, Bruner (2009) introduces another method where only the potential high outcomes of lotteries vary (SGhigh method). Although not present in the literature, we chose to include a method where the potential low outcome varies for reasons of completeness (SGlow method).^10^

#### Paired-gamble methods

Holt and Laury (2002, 2005) introduced the most-cited elicitation method under EUT up to now, where potential outcomes are held constant and the respective probabilities change (PGp). Drichoutis and Lusk (2012, 2016) suggest a similar framework where the outcomes of different lotteries change while the probabilities are held constant. We differentiate these methods further into PGhigh and PGlow depending on whether the high or the low outcome is varied in the MPL. Additionally, the PGall method varies both the probabilities and the potential earnings at the same time.

Many risk elicitation tasks used in the literature fit into the framework of choosing between different lotteries. Sabater-Grande and Georgantzis (2002) provide ten discrete options with different probabilities and outcomes to choose from. Lejuez et al. (2002) introduce the Balloon Analogue Risk Task where subjects could pump up a balloon, and their earnings depend on the final size of the balloon. The larger the balloon gets, the more likely it will explode, in which case the subject earns nothing. Visschers et al. (2009) and Andreoni and Harbaugh (2010) use a pie chart for probabilities and a slider for outcomes to visualize a similar trade-off effect in their experiment. Crosetto and Filippin (2013) present their Bomb Risk Elicitation Task with an interesting framing which quantifies the aforementioned trade-off between probability and potential earnings with the help of a bomb explosion.^11^

#### Questionnaire methods

In addition to the MPL methods, we chose to also incorporate questionnaire risk elicitation methods into our study. Several methods have been introduced that evaluate risk preferences with non-incentivized survey-based methods, and the questions and the methodology they use are very similar. The most recently published ones include the question from the German Socio-Economic Panel Study (Dohmen et al. 2011) or the Domain-Specific Risk-Taking Scale (DOSPERT) by Blais and Weber (2006). For a more detailed description, see the last paragraph of Section 2.

#### Comparison studies

The question arises of which method to use if there is such a large variety of risk elicitation methods and whether they lead to the same results. Comparison studies exist, but the majority compare two methods with each other, and thus their scope is limited. The question of within-method consistency has been addressed by some papers: Harrison et al. (2005) document high re-test stability of the method introduced by Holt and Laury (2002, PGp). Andersen et al. (2008b) test consistency of the PGp (2002) method within a 17-month time frame. They find some variation in risk attitudes over time, but do not detect a general tendency for risk attitudes to increase or decrease. This result was confirmed in Andersen et al. (2008a). Yet there is a gap in the academic literature on the time stability of different methods and their representation that we are eager to fill.

Interestingly, more work has been done on the field of between-method consistency. Fausti and Gillespie (2000) compare risk preference elicitation methods with hypothetical questions using results from a mail survey. Isaac and James (2000) conclude that risk attitudes and relative ranking of subjects is different in the Becker-DeGroot-Marschak procedure and in the first-price sealed-bid auction setting. Berg et al. (2005) confirm that assessment of risk preferences varies generally across institutions in auction settings. In another comparison study, Bruner (2009) shows that changing the probabilities versus varying the payoffs leads to different levels of risk aversion in the PG tasks. Moreover, Dave et al. (2010) conclude that subjects show different degrees of risk aversion in the Holt and Laury (2002, PGp) and in the Eckel and Grossman (2008, SGall) task. Their results were confirmed by Reynaud and Couture (2012) who used farmers as the subject pool in a field experiment. Bleichrodt (2002) argues that a potential reason for these differences might be attributed to the fact that the original method by Eckel and Grossman (2008) does not cover the risk seeking domain, which can be included with the slight modification we made when incorporating this method. Dulleck et al. (2015) test the method devised by Andreoni and Harbaugh (2010) using a graphical representation against the PGp and describe both a surprisingly high level of within- and inter-method inconsistency. Drichoutis and Lusk (2012, 2016) compare the PGp method to a modified version of it where probabilities are held constant. Their analysis reveals that the elicited risk preferences differ from each other both at the individual and at the aggregate level. Most recently, Crosetto and Filippin (2016) compare four risk preference elicitation methods with their original representation and framing and confirm the relatively high instability across methods.

In parallel, a debate among survey-based and incentivized preference elicitation methods emerged which were present since the survey on questionnaire-based risk elicitation methods by Farquhar (1984). Eckel and Grossman (2002) conclude that non-incentivized survey-based methods provide misleading conclusions for incentivized real-world settings. In line with this finding, Anderson and Mellor (2009) claim that non-salient survey-based elicitation methods and the PGp method yield different results. On the contrary, Lönnqvist et al. (2015) provide evidence that the survey-based measure, which Dohmen et al. (2011) had implemented, explains decisions in the trust game better than the SGsure task. Charness and Viceisza (2016) provide evidence from developing countries that hypothetical willingness-to-risk questions and the PGp task deliver deviating results.

#### Further considerations

A recent stream of literature broadens the horizon of investigation to theoretical aspects of elicitation methods: Weber et al. (2002) show that people have different risk attitudes in various fields of life, thus risk preferences seem to be domain-specific. Lönnqvist et al. (2015) document no significant connection between the HLp task and personality traits. Dohmen et al. (2010) document a connection between risk preferences and cognitive ability, which was questioned by Andersson et al. (2016). Hey et al. (2009) investigate noise and bias under four different elicitation procedures and emphasize that elicitation methods should be regarded as strongly context specific measures. Harrison and Rutström (2008) provide an overview and a broader summary of elicitation methods under laboratory conditions, whereas Charness et al. (2013) survey several risk preference elicitation methods based on their advantages and disadvantages.

In addition, there is evidence that framing and representation matters. Wilkinson and Wills (2005) advised against using pie charts showing probabilities and payoffs as human beings are not good at estimating angles. Hershey et al. (1982) identify important sources of bias to be taken into account and pitfalls to avoid when designing elicitation tasks. Most importantly, these include task framing, differences between the gain and loss domains and the variation of outcome and probability levels. Von Gaudecker et al. (2008) show that the same risk elicitation methods for the same subjects deliver different results when using different frameworks — e.g. multiple price list, trade-off method, ordered lotteries, graphical chart representation, etc. This procedural indifference was confirmed by Attema and Brouwer (2013) as well, which implies that risk preferences on an individual level are susceptible to the representation and framing used.

The previous paragraphs lead us to the conclusion that methods should be compared to each other by using the same representation and format. This justifies our decision to compare them using the standard MPL framework which guarantees that the differences cannot be attributed to the different framing and representation of elicitation tasks. However, this comes at the price that we had to change some of the methods slightly, which implies that they are not exactly the same as their originally published versions. We certainly do not claim that the MPL is the only valid framework, but our choice for it seems justified by its common usage and relative simplicity. We consider a future investigation using a different representation technique as a potentially interesting addition. Also, we emphasize that the differences in our results exist among the MPL representations of the methods and they can only be generalized to the original methods to a very limited extent. See Table 3 for an overview of the link between the MPL representation and the particular method that was published originally, and Table 12 in Appendix A, where we compared the results from our MPL methods to the results in previous studies — most of the studies deliver significantly different results to the risk parameters measured in our study. This is not surprising given the considerations in Sections 1.2.4 and 1.2.5, as we map all methods to the MPL space. Furthermore, risk elicitation methods are very noisy in general. For example the same method with the same representation delivers significantly different results in Crosetto and Filippin (2013) and Crosetto and Filippin (2016).

**Table 3:** Link between MPL representation and literature

Method	Corresponding Literature
SGp	Bruner (2009)
SGhigh	Bruner (2009)
SGlow	
SGsure	Cohen et al. (1987), Abdellaoui et al. (2011)
SGall	Binswanger (1980), Eckel and Grossman (2008)
PGp	Holt and Laury (2002), Holt and Laury (2005)
PGhigh	Drichoutis and Lusk (2012, 2016)
PGlow	Drichoutis and Lusk (2012, 2016)
PGall	Sabater-Grande and Georgantzis (2002), Lejuez et al. (2002),
	Andreoni and Harbaugh (2010), Crosetto and Filippin (2013)
Questionnaire	Weber et al. (2002), Dohmen et al. (2011)

Notes: On the left, this table lists all MPL and questionnaire methods, and on the right the corresponding literature.

## Design

We provide a laboratory experiment to compare different MPL risk elicitation methods. Subjects answered the risk elicitation questions first. Then, benchmark games were presented to them to gauge predictive power, which was followed by a non-incentivized questionnaire. We will provide a detailed description on the exact procedures of each part in the later paragraphs.

We conducted ten sessions at the Vienna Center for Experimental Economics VCEE) with 96 subjects.^12^ Sessions lasted about 2 hours, with a range of earnings between 3€ and 50€, amounting to an average payment of 20.78€ with a standard deviation of 10.1€. We calibrated these payments similarly to previous studies (e.g. Bruner (2009) or Abdellaoui et al. (2011), among others). Average earnings were about 9.5€ in the risk task and about 8.3€ in the benchmark games plus a 3.00€ show-up fee. Harrison et al. (2009) provide evidence that the existence of a show-up fee could lead to an elevated level of risk aversion in the subject pool. In our experiment, this moderate show-up fee was only pointed out to the subjects after making their decisions in the risk elicitation methods and the benchmark games. Thus, it could not have distorted their preferences. The experiment was programmed and conducted with the software z-Tree (Fischbacher 2007), and ORSEE (Greiner 2015) was used for recruiting subjects.

We employed a within-subject design, meaning that each subject took decisions in each and every task as in other comparison studies (Eckel and Grossman 2008; Crosetto and Filippin 2016). This property rules out that the methods differ due to heterogeneity between subjects, but it comes with the drawback that methods which were encountered later might deliver more noisy or different results due to fatigue or other factors, as the answer to a particular method could also be a function of previously seen MPLs. Consequently, we included the order in which a method appeared in all regressions as controls wherever possible, compared the variance in earlier and latter methods and tested for order effects; no significant effects were found.^13^ To avoid biases, a random number generator determined the order of methods for each subject separately in the beginning of each session.^14^

After receiving instructions on screen and in written form, subjects went through the nine incentivized risk elicitation methods. In order to avoid potential incentive effects mentioned by Holt and Laury (2002), the expected earnings for a risk-neutral individual were equal in every method. Furthermore, to avoid potential biases due to the different reactions to gains and losses (Hershey et al. 1982), each of our lotteries is set in the gains domain. Andersen et al. (2006) confirmed previous evidence (Poulton 1989) that there is a slight tendency of anchoring and choosing a switching point around the middle for risk elicitation tasks. In order to counteract anchoring and one-directional distortion of preferences as a consequence of this unaviodable pull-to-center effect, each risk elicitation task appeared randomly either top-down or bottom-up. Depending on randomization, out of nine potential switching opportunities the fourth or the sixth option were the risk-neutral switching points.^15^

Subjects also had the opportunity to look at their given answer and modify it right after each decision if they wished to do so. After making a decision in each method, we asked subjects the following question: “On a scale from 1 to 10, how difficult was it for you to make a decision in the previous setting?” With this question we assessed self-perceived complexity of the tasks, since there is evidence in the literature (Mador et al. 2000) that subjects make noisier decisions if the complexity of a lottery increases, and therefore a less complex method is preferred. Moreover, Dave et al. (2010) outline the trade-offs between noise, accuracy and subjects’ mathematics skills. They suggest that it is a good strategy to make MPL tasks simpler for subjects. In this spirit, we asked our subjects to indicate the row in which they switched from the “LEFT” column to the “RIGHT” column, thereby enforcing a single switching point (SSP). Using this framework, subjects were not required to make a decision for each and every row in every method, which would have meant more than 100 monotonous, repetitive binary choices throughout the experiment. Additionally, this approach ensures that the subjects were guaranteed to give answers without preference reversals. We consider this option more viable than accepting multiple switching points — thus allowing inconsistent choices — and using the total number of “safe” choices to determine a subject’s risk coefficient interval. The SSP has been used several times, e.g. Gonzalez and Wu (1999) or Jacobson and Petrie (2009).

By enforcing a SSP, we faced a trade-off between potential boredom and the non-detection of people with inconsistent preferences. Furthermore, some of the reported within-method instability might stem from “fat preferences” or indifference between two or more options. However, the SSP can further be justified in that only a small proportion of subjects expressed multiple switching points in earlier studies,^16^ so this design choice is highly unlikely to drive our results.

In order to test within-method consistency, three of the nine methods were randomly chosen and presented to subjects again, without telling them that they had already encountered that particular method.^17^ Repeating all methods was not feasible due to fatigue concerns, as the experiment is already quite long. This approach allows us to test both within-method and inter-method consistency. The modification of subjects’ answers was allowed here once as well. The perceived complexity of tasks was also elicited again.

Control questions were used for the preference elicitation methods and for each benchmark game in order to verify that subjects understood the task they were about to perform.^18^ Subjects had to answer them correctly in order to participate in the experiment.

We incorporated the random lottery incentive system emphasized by Cubitt et al. (1998). Thus, the computer chose one of the twelve risk preference methods and one of the eight rows within that particular method on a random basis to be payoff-relevant. Additionally, one of the three benchmark games was chosen to be payoff-relevant as well. This random lottery incentive system helps keep the costs at a reasonable level while having similarly sized stakes (than e.g. Bruner 2009) or even larger stakes (than e.g. Holt and Laury 2002 or Harrison et al. 2007) for the elicitation tasks compared to previous studies, while mitigating potential income effects. Nevertheless, we note that the random lottery incentive system might be a potential caveat in our study, since Cox et al. (2015) document somewhat different behavior under various payment mechanisms.

As far as hedging behavior is concerned, Blanco et al. (2010) provide evidence that hedging and the corresponding biased beliefs and actions can only be problematic if the hedging opportunities are highly transparent. Taking this consideration into account, we provided feedback on the outcome of the risk elicitation tasks only at the end of the experiment. Thus, it was not possible for subjects to create a portfolio and use hedging behavior over different parts of the experiment.

On top of the risk elicitation tasks, we used three benchmark games resembling real-life situations as well as situations relevant to economists. As behavior in these settings should only depend on risk attitudes, they will serve as benchmarks to contribute to the debate which risk elicitation methods are appropriate to predict behavior in these games. The benchmark games appeared in a randomized order. First, we used the same investment task as Charness and Gneezy (2010). Here, subjects could decide how much they wanted to invest in stocks and bonds out of an endowment of 10€. Subjects knew that any investment in bonds is a safe investment, and therefore they received the same amount they had invested in bonds as income. Additionally, the amount they invested in stocks was to be multiplied by 2.5 or lost completely with equal chance. Under EUT, this setting implies that both risk neutral and risk seeking decision makers should invest the entire amount. Thus, in order to be able to differentiate between them, we introduced another investment setting where the potential payment for stocks was 1.5 times the invested amount.

The third benchmark game was a first-price sealed-bid auction against a computerized opponent in line with Walker et al. (1987). Subjects could bid between 0.00€ and 20.00€ of their endowment, and they knew that the computer bid any amount between 0.00€ and 20.00€ with equal chance. The potential earnings ( *E*_1_ for subject 1) according to the bids ( *x*_1_, *x*_2_) are:

$$E_{1}= \left\{\begin{array}{ll} 20-x_{1}&\text{if}\,\,x_{1}>x_{2}\\ 0&\text{if}\,\,x_{1}<x_{2}\\ 20-x_{i}\,\,\text{or}\,\,E_{1} = 0\,\,\text{(with 50\% chance)}&\text{if}\,\,x_{1}=x_{2} \end{array}\right. $$

Our benchmark games are deliberately chosen in such a way that risk is clearly relevant in the games, while being one step away from the artificial risk elicitation mechanisms. Therefore, all benchmark games are framed heavily, while still ensuring that risk attitudes should be the only factor driving a subject’s decisions. The investment settings are very similar to the risk elicitation mechanisms described above in the sense that they resemble an SG method (with the difference that you choose your sure payoff and your lottery at the same time). The auction is more complex, as the optimal risk-neutral solution is harder to compute, but here you basically choose your own lottery, too. We therefore expect stronger correlation with the MPL methods for the investment games.

The experiment concluded with an extensive questionnaire. In order to incorporate survey-based measures, we asked subjects to provide an answer on a ten-point Likert-scale to the following two questions in line with Dohmen et al. (2011): “In general, are you a person who is fully prepared to take risks or do you try to avoid taking risks?” and “In financial situations, are you a person who is fully prepared to take risks or do you try to avoid taking risks?” The perceived complexity of these questions was elicited as well. In the questionnaire, we elicited the following socioeconomic factors: Age, gender, field of study, years of university education, nationality, high school grades in mathematics, monthly income and monthly expenditure. Furthermore, we elicited cognitive ability by conducting a cognitive reflection test (Frederick 2005). Lastly, we assessed subjects’ personalities in line with Rammstedt and John (2007), who provide a short measure of personality traits according to the BIG5^19^ methodology introduced by Costa and McCrae (1992).

## Results

We will first establish in Sections 3.1 and 3.2 that the elicited risk parameter is highly dependent on the particular variant of MPL used because the overall distributions of switching points are very diverse and the rank correlations between the different methods are low in most circumstances. Section 3.3 analyzes the common features across methods. In Section 3.4 we apply multiple measures to determine method quality. To this end, we first use benchmark games to let the data speak which risk elicitation methods predict behavior in these games best. In Section 3.4.2, we will show which method produces the most stable results overall. Section 3.5 concludes with the result that the PGhigh method is the most stable method and that it has the highest predictive power.

### Overall distributions are different

According to EUT, a subject’s behavior does not depend on which parameters are changed from row to row, as his underlying risk parameter value is constant. As the different versions of the MPL are calculated in such a way that the same switching point implies the same risk parameter interval, a consistent individual should have the same switching point in all versions of the MPL. This implies that the distributions of switching points should be the same across methods, barring some noise.

**Fig. 1:**
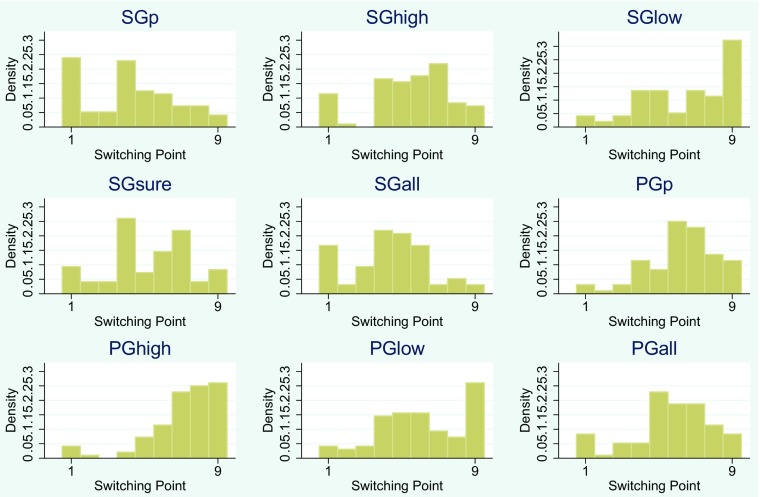
Distributions of risk preferences; a low value indicates risk loving and a high value indicates risk averse behavior; x-axis: switching points (e.g. risk preferences) of subjects, where 1 means a subject switches from left to right in the first row and 9 means a subject never switches; y-axis: frequency of switching point

First, see Fig. 1 for a graphical representation of the distributions. It is clearly visible at first glance that the distributions are not the same across all methods. For example, in the SGp method, most subjects would be classified as highly risk loving, whereas in the PGhigh method the majority of subjects would be classified as risk averse.

To verify whether distributions across methods are the same, we conduct two tests: a Friedman test, which shows that the means are not the same across methods ( *p*<0.0001), and a Kruskal-Wallis test, which shows that the distribution of answers is not the same across methods ( *p*<0.0001). We conclude that the switching points are, contrary to standard theory but in accordance with the literature, dependent upon the version of the particular MPL variation used.

To see which specific versions are significantly different from each other, we conduct a series of Wilcoxon tests, the natural pairwise analogue to the Kruskal-Wallis test. We use the Wilcoxon test to give a comparison of the distributions, as a difference in distributions is a more meaningful statistic here than a comparison of means. The p-values of the pairwise tests can be found in Table 4. Out of 55 pairwise comparisons, 28 comparisons indicate that methods are different at *p*<0.001. Thirty-four (43) instances suggest that methods are different at *p*<0.01 (0.05) significance levels.^20^ To make sure that the differing results are not a product of fatigue or order effects, we also test whether CRRA-coefficients of methods that are encountered later in the experiment exhibit biases or more noise; the resulting tests show no significant order effects overall and across methods.^21^

**Table 4:** Pairwise Wilcoxon test for equality of distribution

	SGp	SGhigh	SGlow	SGsure	SGall	PGp	PGhigh	PGlow	PGall	GQ
SGhigh	.00***									
SGlow	.00***	.00***								
SGsure	.00***	.37	.00***							
SGall	.79	.00***	.00***	.01**						
PGp	.00***	.01**	.28	.00***	.00***					
PGhigh	.00***	.00***	.02*	.00***	.00***	.00***				
PGlow	.00***	.02*	.23	.01**	.00***	.68	.00***			
PGall	.00***	.31	.02*	.04*	.00***	.08	.00***	.39		
GQ	.02*	.03*	.00***	.29	.04*	.00***	.00***	.00***	.01**	
FQ	.00***	.64	.01**	.29	.00***	.02*	.00***	.04*	.36	.00***

Notes: p-values of pairwise Wilcoxon tests are displayed; GQ: general question; FQ: financial question; stars are given as follows: *: p <0.05; **: p <0.01; ***: p <0.001

We conclude that different methods deliver significantly different results, and that the different versions of the MPL cannot be used interchangeably, as the estimated risk preference parameter depends heavily on the version used. Subjects can easily be classified as risk loving in one version and as risk averse in another. Of course we do not know a subject’s true risk preferences, and therefore any of the methods might be able to classify a subject correctly. To provide an answer to this puzzle, see Section 3.4.2, where we conduct a quality assessment of the different methods.

### Rank correlations are low

In this section we look at the rank correlation coefficients between the different methods and the questionnaire answers. If there are high rank correlations between the risk elicitation methods, one might argue that it is irrelevant which one is used if one intends to control for risk attitudes under any given circumstance. Rank correlations between the MPL methods and the questionnaire measures can be found in Table 5. We see that some of the correlations are significant, but only 11*%* of all pairwise comparisons in total if we test conservatively at *p*<0.001 because of the multiple testing problem. Pay special attention to the fact that PGp, the most widely used method today, has no significant rank correlations with any of the other methods.^22^ See also Table 10 in Appendix A for standard correlations, which basically gives the same results as Table 5.

These findings provide further evidence that the elicitation procedure should be chosen with care as the elicited risk aversion coefficient and also the relative ranking of subjects according to each method varies within broad boundaries.

### Method similarities

We have established in Sections 3.1 and 3.2 that there are significant differences in the distributions of the risk elicitation methods. There are, however, some similarities that can be observed across methods: In Table 6, we classify MPLs according to whether the high payoff, the low payoff, the probabilities or the certainty equivalents change in the MPL table, whether the method has a certainty equivalent and whether the table was presented in a top-down or bottom-up format. Furthermore, we control for age, gender, cognitive reflection scores and the order in which the tables were presented. Column 1 shows the results for the first time a method was encountered, and column 2 for the repeated measurements.

**Table 6:** Similarities across all methods

	Not Repeated	Repeated
High Payoff changes	−.108 ^∗∗^	.052
Low Payoff changes	.208 ^∗∗∗^	.206 ^∗∗∗^
Probability changes	−.306 ^∗∗∗^	−.335 ^∗∗∗^
Certainty Equivalent changes	.086 ^∗^	−.039
Has Certainty Equivalent	−.496 ^∗∗∗^	−.504 ^∗∗∗^
Top-Down Representation	.085 ^∗∗^	.035
Constant	.835	.205
*R* ^2^	.121	.190
Number of Observations	864	288

Notes: OLS regressions clustered by individual subjects with one observation being the outcome from one answer of one subject in one method; dependent variable is the resulting CRRA-coefficient, with low scores indicating risk-loving behavior; independent variables on the left are dummies; nonsignificant controls for age, gender, order, BIG5 scores, income and CRT scores are included in the regressions but omitted in the table; first column gives results for the first time subjects encountered one of the nine methods, second column for the repeated measurements; stars are given as follows (differently than in the other tables, due to the absence of a multiple testing problem): *: p <0.10; **: p <0.05; ***: p <0.01

We see that generally, methods that change the probabilities or methods that have a certainty equivalent classify subjects as more risk loving, while methods that change the low payoffs classify subjects as more risk-averse.^23^ When a method is presented to subjects for the first time, changing the high payoff also classifies them as more risk-loving, while presenting the table with ascending numbers seems to classify subjects as more risk-averse, although these two effects seem to vanish when presenting subjects with the same tables again. Note that we do not observe order effects, or significant effects of the control variables.

### Method quality indicators

We use two avenues to measure a method’s quality: its predictive power (Section 3.4.1) and its stability (Section 3.4.2).

#### Predictive power

In order to see which method predicts behavior best in our benchmark games, we look at three statistics: the predictive power by simple OLS regression, the predictive power by Spearman rank correlation, and the absolute average deviation from the prediction.

In Table 7, we see the outcome of OLS regressions in the upper part, while controlling for personality measures and socioeconomic variables. In the lower part of Table 7 you see Spearman rank correlation coefficients, which we include because besides the absolute size of the elicited coefficients, the correct rank ordering of subjects is essential since these methods are often used to control for the role of risk attitude in various settings. The OLS regression can be understood as follows: The dependent variable is the outcome of a particular benchmark game, and the independent variables include the outcome in terms of the elicited risk aversion parameter *ρ* of one of the risk elicitation methods^24^ plus all controls mentioned above.^25^ The resulting coefficients in the investment games are negative because a higher *ρ* implies risk-averse behavior, and therefore lower investments and bids in the benchmark games; the reverse is true for the auction. The corresponding adjusted *R* ^2^ values can be found in parentheses below the coefficients.

The OLS regression equation is then given by

$$\begin{array}{@{}rcl@{}} BG_{i,j}=\beta_{0}+\beta_{1}*MPL_{j}+\sum\limits_{k=2}^{6}\beta_{k}*BIG5_{k}+\sum\limits_{l=7}^{11}\beta_{l}*SE_{l}+\beta_{12}*CRT+\epsilon_{i}\text{,} \\ \end{array} $$

where *i* denotes the index of benchmark games (*BG*), *j* denotes the index of risk measures, *MPL* denotes the outcome of a risk elicitation method, *BIG5* denotes personality measures according to the *BIG5*, *SE* denotes the socioeconomic variables and *CRT* denotes the number of correct answers in the cognitive reflection test.

Additionally, we can calculate a point prediction in each of the benchmark games for each risk elicitation method (but not for the questionnaires). In Table 8 we report the absolute average deviations from these predictions, averaged over all three benchmark games according to the formula

$$\begin{array}{@{}rcl@{}} AAD=\left(\sum\limits_{i=1}^{n} |H_{i}-H_{i}^{*}|+\sum\limits_{i=1}^{n} |L_{i}-L_{i}^{*}|+\sum\limits_{i=1}^{n} \frac{|A_{i}-A_{i}^{*}|}{2}\right)/(3n), \\ \end{array} $$

where *H*_*i*_ denotes high investment game outcomes and $H_{i}^{*}$ high investment game predictions (*L* stands for investment low and *A* for auction).^26^

In the auction, none of the methods produce statistically significant results in the OLS regression. This is puzzling, as the auction can in itself be seen as a risk elicitation procedure, albeit with heavy framing. Recent literature, however, provides evidence that not only risk attitudes but also other factors like regret aversion (Engelbrecht-Wiggans and Katok, 2008) could drive behavior in auctions. As far as the Spearman rank correlation is concerned, the SGp method is the only one that is rank correlated (*p*<0.05) with auction behavior.

In the investment games, the methods produce much better results. In the low investment setting, PGhigh has the biggest explanatory power, with SGhigh being a close follower. Note that it is surprising that PGhigh is the best predictor both in the regression and the rank correlation, as the investment games in themselves can be interpreted as standard gamble methods, so one would expect one of these methods to perform best.

In the high investment setting, many methods (PGhigh, PGlow, SGhigh, SGlow, SGp, and the questionnaires) are able to explain a part of the variance, with SGp being the one giving the best results (*p*<0.01). Note that in this setting, survey-based measures perform very well, so questionnaire measures seem to serve as good proxies for subjects’ risk preferences in some circumstances. Note that the adjusted *R* ^2^ values are relatively low in general; we added the above mentioned controls to our regressions, which are not able to pick up much of the variation.^27^

As far as the deviations from the predictions are concerned, PGhigh performs best with an average deviation of 1.75 across all benchmark games with SGp and SGlow also having low deviations.^28^

In conclusion, PGhigh and SGp yield the best results in explaining behavior, with PGhigh having the lowest deviation from the prediction of behavior in the benchmark games. We conclude that PGhigh has the highest predictive power with SGp being a close runner-up. Additionally, we relax our assumptions on CRRA and perform robustness checks taking CARA, DRRA, DARA, IRRA and IARA into account in Tables 23–34 in the Online Resource.^29^ Furthermore, due to the ample evidence on the violations of EUT, we provide the same regressions by taking probability weighting,^30^ prospect theory^31^and cumulative prospect theory into consideration.^32^ The results show that our findings remain quantitatively and qualitatively the same under different specifications. In general, we see similar explanatory power and in the vast majority of cases the same significance levels for the PGhigh and SGp methods, which confirm our findings. In some specifications, we even see that the coefficient for PGhigh becomes significant also for the investment games with low stakes, for example under DRRA. Nevertheless, a further justification is that some of the other methods (SGhigh, PGlow and questionnaire methods) lose significance (for example under PT, IRRA or IARA) under some of the above mentioned alternative theoretical foundations and functional forms.

#### Stability measures

In this section, we evaluate the stability of the different MPL representations. Remember that after our subjects had gone through all nine MPL methods, three of them were randomly chosen and presented to them again. A method can be described as stable if the given answers between the first and the second time a method was encountered are very similar. To analyze this similarity, we use three criteria: equality of overall distribution, equality of rank ordering and absolute average deviation between the first and second answers. For reasons of completeness, we also report the perceived complexity of each method.^33^

Table 9 reports these measures. In the first column we give p-values from a Kolmogorov-Smirnov test that evaluates whether the distributions of the first and the second time a method is encountered are the same. A significant p-value means that the distributions are significantly different from each other, indicating a low stability of overall distribution across a 30 minute time period.

**Table 9:** Stability Measures

Method	KS-Test	Rank Corr.	AAD	Complexity
SGp	.453	.51***	1.60	3.42
SGsure	.003	.51***	1.37	3.92
SGhigh	.644	.39**	1.48	3.97
SGlow	.007	.35	1.96	3.20
SGall	.005	.16	1.8	4.81
PGp	.240	.23	1.33	4.21
PGhigh	.879	.45***	1.24	3.78
PGlow	.006	.25	2.04	4.29
PGall	.000	.19	1.85	5.75

Notes: First column: P-values for a Kolmogorov-Smirnov test of equality of distributions; Second column: Rank correlation between the distributions of first and second answers (stars indicate significant rank correlation); Third column: Absolute average deviation (AAD) between the first and the second decision in the same method; Fourth column: Indicates a subject’s perceived complexity of a method; Stars are given as follows: *: p <0.1; **: p <0.05; ***: p <0.01

The second column gives the rank correlation between the first and second time a method was encountered. This measure is important because if a method’s overall distribution merely shifted up or down without changing the rank ordering of subjects, this method can also be described as stable since the ordering of subjects remains the same.

The third column reports the absolute average deviation (AAD) of subjects’ answers when a particular method is presented to them again, compared to the first time — a lower value is therefore better. The last column gives the means of the perceived complexity of a method on a 1 to 10 Likert scale.

**Fig. 2:**
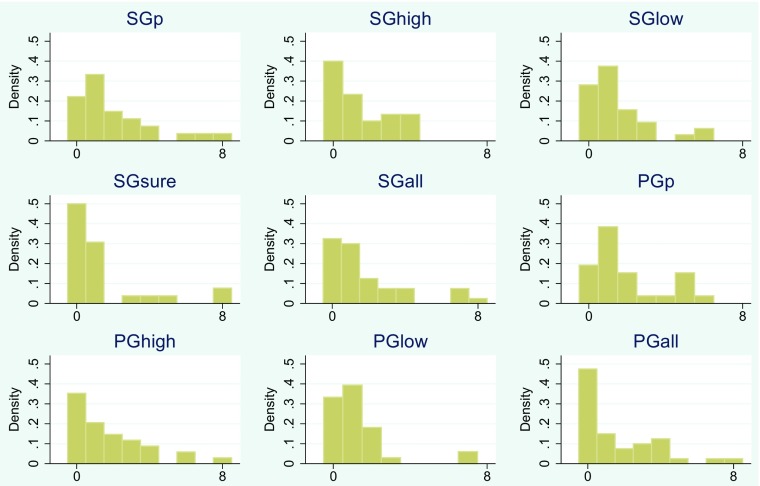
Distributions of absolute differences in switching points between the first and the second time a method is encountered

To visualize these results, we also report the distributions of the differences in switching points between the first and the second time a method is encountered in Fig. 2.

Any method that does not yield stable results over a 30 minute time period cannot be described as stable, and stability is a highly preferable characteristic in a risk measure. For the KS-test (column 1 in Table 9), stability relative to the other methods is indicated by a nonsignificant result: For any methods with a significant result, the overall distributions of answers are different between the first and the second time a method was encountered. Four of the methods have a nonsignificant p-value: SGp, SGhigh, PGp, PGhigh.

A significant rank correlation (column 2 in Table 9) also indicates a stable risk measure, indicating a shift in the distribution, but no change in rank ordering. We see that three of those four methods have significant rank correlations with *p*<0.01: SGp, SGsure and PGhigh.

A low absolute average deviation in answers is also an indicator of a stable risk measure, and the method with the lowest deviation is PGhigh, followed by PGp and SGsure.

Concerning the complexity, we see that a method that is perceived as less complex does not necessarily imply more stability in answers, as SGlow has the lowest complexity rating, yet it is classified as unstable in all three categories. However, a general tendency of low complexity indicating more stability can be observed.

As far as a possible relationship between stability and the control variables (CRT and BIG5 scores, age, gender, income, years of university education) is concerned, no significant effects have been found, so the results will be omitted here.^34^ Finally, we mention that the slight differences in the number of observations between the repetitions of particular methods — caused by the pseudo-random number generator — do not drive within-method consistency.

We conclude that PGhigh is the most stable method, as it is the only method that performs well in all three categories, with the overall distributions of switching points not being significantly different, high rank correlations and low average deviation. SGp, SGsure and PGp perform well in two of the three categories.

### Results conclusion

In the benchmark games, as far as predictive power is concerned, we conclude that PGhigh has the highest predictive power with SGp being a close second, irrespective of the assumed functional form or theoretical framework.^35^ We conclude that only the PGhigh (Drichoutis and Lusk, 2012 & 2016), PGp (Holt and Laury 2002), SGsure (Cohen et al. 1987; Abdellaoui et al. 2011) and SGp (Bruner 2009) methods lead to consistent results within a 30-minute time frame, with the PGhigh method being by far the most consistent: The PGhigh method’s performance is superior to the other methods in terms of deviations from normative predictions, overall and relative stability across time, etc. Our findings are further supported by the fact that we controlled for personality traits, order effects, various socioeconomic factors and cognitive reflection in our analyses.

Therefore, we conclude that while SGp also has high predictive power and good stability in answers, the most stable MPL method with the highest predictive power is PGhigh, which corresponds to a method derived by Drichoutis and Lusk (2012, 2016) in our alternative interpretation.

## Conclusion

We conducted a holistic assessment and analysis of MPL risk elicitation methods that are present in the economics literature with a sophisticated experimental design using a unified framework and representation method. Previous findings in the literature (Dave et al. 2010; Crosetto and Filippin 2016; etc.) indicate that between-method consistency of particular methods is low. We confirm this finding by extending our analysis to all popular methods using the same representation. Furthermore, we show that distributional differences among methods are far from negligible. In addition, we investigate the time consistency of all these methods and document substantial differences in a 30-minute time period for most of the methods. All this implies that an arbitrary selection of a particular risk assessment method can lead to differing results and misleading revealed preferences. Thus, it matters which elicitation method is used by researchers in order to control for risk and other preferences.

Our main takeaway is that we provide a suggestion for which elicitation method to use based on objective criteria that assess within-method as well as between-method consistency and validity in real-world settings such as investments and auctions, and our suggestion is to use the PGhigh method by Drichoutis and Lusk (2012). This particular method performs best if we look at the absolute deviations from the normative predictions in benchmark games and also in terms of rank correlations. Furthermore, it yields highly correlated results within a 30-minute time frame in terms of individual deviations and overall distribution. These findings remain robust — in some cases even more pronounced — if we relax our assumptions on CRRA to alternative functions such as CARA, DRRA, DARA, IRRA and IARA. Moreover, our conclusions remain the same if we allow subjective probability weighting or if we estimate risk attitude parameters in line with prospect theory or cumulative prospect theory.

In a broader context, one should take care when choosing which risk elicitation method to use, especially if one aims to control for risk attitudes in potential real-world contexts such as investment into assets. To be taken into consideration are the nature of the task they intend to control for, trade-off effects between noise, exactness and simplicity. Moreover, we find that changing both the potential rewards and probabilities is perceived as relatively complex by subjects and yields inconsistent results. A further point to consider is that varying the potentially achievable minimum payoff seems to induce more risk-averse behavior while the presence of a certainty equivalent fosters risk taking. Cognitive ability, personality traits and other socioeconomic factors do not seem to be related to risk aversion nor to the extent of consistency we measured.

The debate between changing the probabilities or rewards (Bruner 2009) seems to be far from settled as one of the methods in each context (PGhigh and SGp) delivers promising results. In addition, our findings might provide guidance in implementing other elicitation methods in the MPL format — e.g. loss aversion (Gächter et al. 2010), willingness to pay (Kahneman et al. 1990), individual discount rates (Harrison et al. 2002) — in terms of whether to vary probabilities, rewards or to use a certainty equivalent. On a final note we suggest that the relatively high variation in risk preferences across and within particular methods might not be mere artifacts — especially in light of other recent evidence (Andreoni et al. 2015). We encourage further research to shed light on the consistency of other preference elicitation mechanisms such as social preferences or overconfidence.

### Electronic supplementary material

Below is the link to the electronic supplementary material.

(PDF 315 KB)

## Appendix

### A.1 Robustness checks

Table 10 shows standard correlation coefficients between methods; the results are qualitatively the same as in Table 5.

**Table 10:** Correlation coefficients between the methods

	SGp	SGhigh	SGlow	SGsure	SGall	PGp	PGhigh	PGlow	PGall	GQ
SGhigh	.46***									
SGlow	.37***	.46***								
SGsure	.01	.18	.25*							
SGall	.02	.12	0	.16						
PGp	.13	.12	.15	.23**	−.08					
PGhigh	.07	.27**	.10	.26*	.12	.26*				
PGlow	.27**	.21*	.20	.17	−.04	.12	.10			
PGall	.17	.16	−.06	.04	.17	−.01	−.08	.03		
GQ	.12	.16	0	−.14	.09	−.07	.12	.02	.01	
FQ	.25**	.17	.27**	.19	.07	−.05	−.02	.20	.03	.46**

Notes: This table shows standard correlations as opposed to Spearman rank correlations as in Table 5. SG stands for “standard gamble” and PG for “paired gamble”. Our conclusions remain qualitatively the same. Stars are given as follows: *: p <0.05; **: p <0.01; ***: p <0.001

Table 11 tests for linear order effects in the data: As the nine methods were presented to subjects in a randomized order, risk aversion or noise might increase or decrease as a function of previously seen MPLs. Coefficients in Table 11 can be interpreted as a change in standard deviation (i.e. noise) of CRRA coefficients or changes in CRRA coefficients themselves as a function of order, i.e. the number of previously encountered MPLs. No significant effects were found.

**Table 11:** Testing for order effects – No significant effects

Method	Dependent Variable	
	CRRA Standard Deviation	CRRA
PGhigh	−0.001	0.006
PGlow	−0.013	0.019
PGp	−0.021	0.028
PGall	−0.013	0.013
SGhigh	−0.007	−0.014
SGlow	−0.023	0.029
SGsure	0.031	−0.037
SGp	0.021	−0.020
SGall	−0.011	−0.004
Overall	−0.004	0.000

Notes: Reported coefficients for OLS regressions; the dependent variable is the standard deviation (column 1) of the CRRA-coefficient or the CRRA score (column 2) across all subjects in one particular order, the independent variable is the order in which a method appeared, i.e. the number of previously encountered MPLs; stars are given as follows: *: p <0.1; **: p <0.05; ***: p <0.01

For robustness checks on functional form (PT, CARA, DRRA, IRRA, IARA, DARA and probability weighting), please refer to the Online Resource, Tables 23–34.

### A.2 Comparison of our results to the results in previous studies

In Table 12 we see the differences in the mean values of CRRA risk coefficients between previous studies and our results for each method, where we find that several studies deliver significantly different results to ours. This is not surprising for two reasons: First, risk elicitation methods are very noisy in general. For example the same method with the same representation delivers significantly different results in Crosetto and Filippin (2013) and Crosetto and Filippin (2016), or the task by Holt and Laury (2002), which delivered highly heterogeneous results in past studies as Table 12 shows. Second, framing and representation are vastly different in most studies when compared to our study. Furthermore, in the studies by Eckel and Grossman (2008), Reynaud and Couture (2012) and Crosetto and Filippin (2016) the risk loving domain is not covered in the SGall task; that and the pull-to-center effect drives the risk estimates to be higher, which is also suggested by Bleichrodt (2002) and Andersen et al. (2006).

**Table 12:** Comparison of results to previous studies

Method	Our study		Previous Studies				t-test
	Mean	SD	Mean	SD	Subjects	Study	p-value
PGhigh	0.87	0.56	0.35	0.18	100	Drichoutis and Lusk (2012)	.001
PGlow	0.57	0.67	0.35	0.18	100	Drichoutis and Lusk (2012)	.002
PGp	0.62	0.54	0.32	0.41	175	Holt and Laury (2002)	.001
			0.23	0.14	39	Abdellaoui et al. (2011)	.001
			0.59	0.07	100	Drichoutis and Lusk (2012)	.145
			0.39	0.54	78	Dulleck et al. (2015)	.006
			0.43	0.6	444	Crosetto and Filippin (2016)	.004
			0.62	0.8	268	Andersen et al. (2008b)	1
			0.67	0.57	881	Dave et al. (2010)	.455
PGall	0.47	0.62	0.82	−	86	Lejuez et al. (2002)	−
			1.13	0.64	444	Crosetto and Filippin (2016)	.001
			0.7	0.83	444	Crosetto and Filippin (2013)	.011
SGhigh	0.4	0.65	0.51	0.59	157	Bruner (2009)	.168
SGlow	0.69	0.68					
SGsure	0.31	0.66	0.2	0.08	39	Abdellaoui et al. (2011)	.302
SGp	0.02	0.72	0.45	0.45	157	Bruner (2009)	.001
SGall	0.07	0.63	0.6	0.59	256	Eckel and Grossman (2008)	.001
			0.73	0.9	30	Reynaud and Couture (2012)	.001
			0.694	0.33	444	Crosetto and Filippin (2016)	.001

Notes: mean and standard deviation in terms of CRRA-coefficients; *N*=96 in our study; PGp by Crosetto and Filippin (2016) follows the method in Lejuez et al. (2002); in Lejuez et al. (2002) no standard deviation was reported

## Footnotes

1
^1^ To ease comparison to existing studies, we used exactly the same coefficient intervals as Holt and Laury (2002).

**Table 1:** Risk parameter intervals (Holt/Laury)

Interpretation	Switching	Risk parameter
by Holt and Laury (2002)	Point	Interval
Highly risk loving	1	*ρ*≤−0.95
Very risk loving	2	−0.95 <*ρ*≤−0.49
Risk loving	3	−0.49 <*ρ*≤−0.15
Risk neutral	4	−0.15 <*ρ*≤0.15
Slightly risk averse	5	0.15 <*ρ*≤0.41
Risk averse	6	0.41 <*ρ*≤0.68
Very risk averse	7	0.68 <*ρ*≤0.97
Highly risk averse	8	0.97 <*ρ*≤1.37
Stay in bed	Never	*ρ*>1.37

Notes: This table indicates the mapping from a subject’s chosen switching point into the resulting risk parameter intervals in each method; the leftmost column contains the interpretation of the risk intervals; “Never” means a subject prefers the option “Left” in each row

2
^2^ $u(c)=\frac {c^{1-\rho }}{1-\rho }$

3
^3^ $u(c)= \left \{\begin {array}{lll} c^{\alpha }&\text {if} \,\,c\geq 0\\-\lambda (-c)^{\beta } & \text {if}\,\, c<0 \end {array}\right .$

4
^4^ Note that this approach is also popular among economists due to its computational ease: The vast majority of economic experiments assumes CRRA as well, which makes our results comparable to theirs.

5
^5^ $u(c)=\frac {-e^{\rho c}}{\rho }$

6
^6^ *u*(*c*,*W*)=[(*ω* *W* ^*r*^+*c* ^*r*^)^(1−*ρ*)/*r*^]/(1−*ρ*)

7
^7^ $u(c,W)=\frac {-e^{-\rho r(c+W \omega )}}{\rho }$

8
^8^ This implies that the same switching point in two methods does not yield the same risk parameter estimate under different specifications, but these estimates are still directly comparable according to theory, as they claim to measure a subject’s underlying risk attitudes ceteris paribus.

9
^9^ For a complete list of all methods with the corresponding parameter values (as presented to subjects), refer to the Online Resource.

**Table 2:** Method overview

	What is changing?			
Method	Probability	Highest payoff	Lowest payoff	Sure payoff
SGp	yes	no	no	no
SGhigh	no	yes	no	no
SGlow	no	no	yes	no
SGsure	no	no	no	yes
SGall	no	yes	yes	yes
PGp	yes	no	no	NA
PGhigh	no	yes	no	NA
PGlow	no	no	yes	NA
PGall	yes	yes	yes	NA

Notes: This table indicates which parameters change from row to row in each method, where SG stands for “standard gamble” and PG stands for “paired gamble.”

10
^10^ For examples, see Tables 13−17 in the Online Resource, which correspond to the SG methods.

11
^11^ For examples, see Tables 18−21 in the Online Resource, which correspond to the PG methods.

12
^12^ One subject has been excluded from our subject pool after repeatedly being unable to answer the control questions correctly.

13
^13^ See Table 11 in Appendix A.

14
^14^ Each subject encountered the methods in a unique random order and each order was used only once in the entire experiment.

15
^15^ An example for the difference between the top-down and bottom-up representation can be found in Table 22 in the Online Resource.

16
^16^ E.g. 8.5% in Dave et al. (2010) and 6.6% in Holt and Laury (2002).

17
^17^ The exact number of occasions a particular method was encountered a second time is roughly equal across methods: PGhigh(33), PGlow(25), PGp(26), PGall(39), SGhigh(30), SGsure(33), SGlow(27), SGp(40), SGall(35).

18
^18^ See the Online Resource for the exact text of these questions.

19
^19^ In the BIG5, personality is measured along five dimensions: Agreeableness, Conscientiousness, Extraversion, Neuroticism and Openness.

20
^20^ Note that one should be careful while reading this table and the ones following because of the presence of the multiple testing problem; therefore, we introduce a new notation in the tables: P-values lower than 0.001 are denoted by three stars, p-values lower than 0.01 are denoted by two stars and p-values lower than 0.05 are denoted by one star. *p*<0.001 can be interpreted as significant, even when using the conservative Bonferroni correction (see Abdi 2007).

21
^21^ See Table 11 in Appendix A.

22
^22^ Also, the financial questionnaire (FQ) results have much higher correlations with the other methods than the general questionnaire (GQ) results, strengthening the argument that risk attitudes are domain specific.

**Table 5:** Spearman rank correlation coefficients

	SGp	SGhigh	SGlow	SGsure	SGall	PGp	PGhigh	PGlow	PGall	GQ
SGhigh	.46									
SGlow	.33***	.44***								
SGsure	.05	.22*	.26*							
SGall	.03	.18	-.03	.19						
PGp	.17	.15	.17	.21*	-.04					
PGhigh	.20	.39***	.21*	.03**	.21*	.25*				
PGlow	.31**	.28**	.25*	.19	-.02	.13	.21*			
PGall	.24	.21*	-.01	.08	.19	.04	-.01	.08		
GQ	.15	.13*	.06	-.12	.11	.02	.14	.04	.06	
FQ	.26*	.23*	.29*	.18	.10	-.04	.04	.24*	.13	.46***

Notes: Table includes the nine different methods and the questionnaires (GQ: general questionnaire, FQ: financial questionnaire); stars are given as follows: *: p <0.05; **: p <0.01; ***: p <0.001

23
^23^ In two MPL methods, PGall and SGall, multiple characteristics of the MPL table were changed at the same time. Consequently, in these methods the effects add up.

24
^24^ The results do not change qualitatively if we use the switching points as independent variables instead of *ρ*. This data is available upon request.

**Table 7:** Explanatory power

	SGp	SGhigh	SGlow	SGsure	SGall	PGp	PGhigh	PGlow	PGall	GQ	FQ
Auction	OLS coefficients										
	.68	.52	.57	−.03	−.3	.03	0	.58	−.52	.13	−.08
	(.05)	(.03)	(.04)	(.02)	(.02)	(.02)	(.02)	(.04)	(.03)	(.03)	(.03)
Inv. Low	−.09	−.94*	−.48	.01	−.67	−.39	−1.48***	−.23	−.44	.3*	.09
	(.00)	(.03)	(.00)	(.00)	(.00)	(.00)	(.08)	(.00)	(.00)	(.02)	(.00)
Inv. High	−.9**	−.68	−.28	.22	.58	−.46	−.66	−.65	.2	.28**	.25**
	(.16)	(.12)	(.09)	(.09)	(.11)	(.09)	(.11)	(.12)	(.08)	(.13)	(.13)
	Auct.										
Auct.	.23*	.09	.14	.17	.07	.11	.06	.16	−.13	.10	.11
Inv. Low	.02	.19	.17	.06	.06	.11	.36***	.12	.04	.19	.11
Inv. High	.28**	.28**	.05	.00	.03	.13	.26**	.23*	.09	.31**	.28**

Notes: In the OLS regression, the dependent variable is the outcome in one of the four benchmark games, the independent variables are the outcome in terms of *ρ* from one method plus controls (age, gender, BIG5, CRT test, income, years of university education); the adjusted *R* ^2^ value for the regression can be found below a coefficient; Stars are given as follows: *: p <0.05; **: p <0.01; ***: p <0.001

25
^25^ It is not possible to add controls in the Spearman rank correlation.

26
^26^ Note that we divide the deviation in the auction game by 2 because the choice range in the auction game is twice as high.

**Table 8:** Deviations from predictions

	SGp	SGhigh	SGlow	SGsure	SGall	PGp	PGhigh	PGlow	PGall
Deviation	1.91	2.41	2.19	2.03	2.11	2.27	1.75	2.17	2.11

Notes: Absolute average deviations from the predictions in the benchmark games

27
^27^ In all regressions, none of the controls were significant at *p*<0.05. This implies that behavior seems to primarily be driven by risk attitudes.

28
^28^ Note that one might be concerned with this analysis because if a method generally classifies subjects as risk-averse, it is not surprising that it explains behavior well in the low investment setting, as subjects naturally behave risk-aversely in this setting due to the parameters. However, this critique is not valid for any method that provides good predictions across multiple benchmark games (e.g. PGhigh).

29
^29^ We used subjects’ self-reported monthly income and expenditure as a proxy for their wealth. This was necessary, since our subject pool consisted of students who are not expected to have any wealth, but their monthly income or expenditure can serve as a good proxy for this purpose as they are expected to be highly correlated with their wealth (Persson and Tabellini 1994) and social class.

30
^30^ In the Online Resource, we report the regressions using the probability weighting function $w(p)=\frac {p^{\gamma }}{[p^{\gamma }+(1-p)^{\gamma }]^{\frac {1}{\gamma }}}$.

31
^31^ As our lotteries are in the gains domain, prospect theory amounts to *u*(*c*)=*c* ^*α*^.

32
^32^ For prospect theory and cumulative prospect theory as well as probability weighting functions, we used the functional form and parameters provided in Tversky and Kahneman (1992) and Quiggin (1982) to create the tables in the Online Resource, different specifications for PT provided in Camerer and Ho (1994) and in Wu and Gonzalez (1996), as well as for PW in Prelec (1998), do not change the results qualitatively. Specifications and parameters for DRRA and IRRA can be found in Andersen et al. (2012) and Saha (1993), respectively, for IARA and DARA in Saha (1993).

33
^33^ We do not use complexity as a stability measure, as the impact of a higher perceived complexity is not clear. On the one hand, one might argue that a higher measure in these categories implies noisier behavior, but on the other hand one might argue that a subject takes more time thinking about the problem at hand.

34
^34^ On the subject level, we tested whether the average deviation between the first and repeated decisions are related to any of the control variables, whether the standard deviation of CRRA scores across all methods is related to the control variables, and whether the average differences between the predicted and the actual outcomes in the benchmark games are related, both per method and overall. At most weakly significant results were found; the results are available from the authors upon request.

35
^35^ For non-incentivized surveys, our data shows that eliciting preferences with general and financial questions is a relatively good predictor compared to several incentivized elicitation methods.
