# Supplementary material for: How to reveal people’s preferences: Comparing time consistency and predictive power of multiple price list risk elicitation methods
Source: J Risk Uncertain. 2017 Feb 1;53(2):107–36. doi: 10.1007/s11166-016-9247-6 (PMC5366177; doi:10.1007/s11166-016-9247-6)
Supplement: Supplementary file 1 — (PDF 315 KB) [file 11166_2016_9247_MOESM1_ESM.pdf]

manuscript No.
(will be inserted by the editor)

How to reveal people's preferences: Comparing time consistency and predictive power of multiple price list risk elicitation methods - Electronic Supplementary Material

Journal of Risk and Uncertainty
Volume 53, Number 2/3

Tamás Csermely ·
Alexander Rabas

Tamás Csermely
University of Vienna, Doctoral School of Operations Management and Logistics
Oskar Morgenstern Platz 1, 1090, Vienna, Austria
Vienna University of Economics and Business, Institute for Public Sector Economics, Vienna, Austria
Lauder Business School, Vienna, Austria
E-mail: csermi@gmail.com

Alexander Rabas
University of Vienna, Doctoral School of Economics Vienna, Department of Economics
Oskar Morgenstern Platz 1, 1090, Vienna, Austria

Instructions

General Instructions

Welcome to this experiment in decision making.

You will be asked to make a series of choices that will affect your payment at the end of the experiment. Please pay close attention to the instructions, and if you have any questions raise your hand and an employee of the lab will help you with any questions you might have.

Also, you will be asked to fill out a short questionnaire, where your answers are not relevant for your payoff. All your decisions and answers during the experiment will stay completely anonymous to everyone.

In 'Part 1', you will make decisions in twelve different situations, where in each situation you will make one choice.

A sample decision screen is provided above and on the piece of paper in

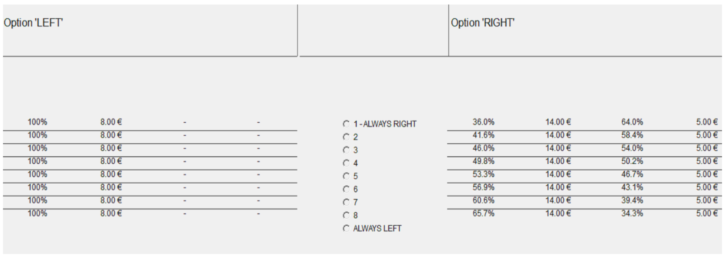

Fig. 3 *This is the first sample screen subjects saw; note that it does not match any of the nine methods.*

front of you. Please feel free to take notes on this paper.

On each screen you see two columns ('LEFT' and 'RIGHT'). In each column you have eight rows with different payment possibilities. You have to decide which option you prefer in each row.

For example on the screen provided above, in the first row you will have to choose between:
'LEFT': receive 8€ with 100% probability
'RIGHT': receive 14€ with 36.0% probability or 5€ with 64.0% probability

In the second row, you would make a choice between:
'LEFT': receive 8€ with 100% probability
'RIGHT': receive 14€ with 41.6% probability or 5€ with 58.4% probability

In the center of the screen you will find a number of radio buttons. You can only click one of those buttons on each screen. This button indicates at what row you want to switch from 'LEFT' to 'RIGHT'.

If you choose row 3, that means you prefer 'LEFT' for the first 2 rows,

Option 'LEFT'				Suppose that your earnings depend on the following lottery.	Option 'RIGHT'			
24.0%	11.36 €	76.0%	0.00 €	1 - ALWAYS RIGHT	23.0%	12.00 €	77.0%	0.00 €
34.0%	9.76 €	66.0%	0.00 €	2	27.0%	11.36 €	73.0%	0.00 €
44.0%	8.16 €	56.0%	0.00 €	3	29.7%	10.93 €	70.3%	0.00 €
54.0%	6.56 €	46.0%	0.00 €	4	32.3%	10.51 €	67.7%	0.00 €
64.0%	4.96 €	36.0%	0.00 €	5	36.6%	9.83 €	63.4%	0.00 €
74.0%	3.36 €	26.0%	0.00 €	6	42.5%	8.88 €	57.5%	0.00 €
84.0%	1.76 €	16.0%	0.00 €	7	56.0%	6.72 €	44.0%	0.00 €
94.0%	0.16 €	6.0%	0.00 €	8	91.7%	1.01 €	8.3%	0.00 €
				ALWAYS LEFT				

Fig. 4 *Example Screen with colorcoding*

but you prefer 'RIGHT' for rows 3-8.
If you choose row 6, that means you prefer 'LEFT' for the first 5 rows, but you prefer 'RIGHT' for rows 6-8.
If you choose row 1 - ALWAYS RIGHT, that means you prefer 'RIGHT' in every row.
If you choose row ALWAYS LEFT, that means you prefer 'LEFT' in every row.

When you are finished making your decision for a screen, click 'OK' and you will get to the next screen where you will see your choice again. In case you are not satisfied with your choice, you can change your choice once if you wish to. Your second choice is final and cannot be changed afterwards.

After each decision screen, we will ask you how difficult it was for you to make a decision on the previous screen. These questions do not affect your payment. Still, we ask you to answer truthfully.

For your payoff of Part 1, one screen of the twelve is randomly selected by the computer. The computer will also select one of the rows at random. In this row, you have chosen 'LEFT' or 'RIGHT'. Finally the computer will randomize between the two possible outcomes based on the given probabilities.

For example, the computer chooses at random the following row to be relevant for your payoff:
'LEFT': 'receive 20€ with 70% probability or 5€ with 30% probability.'
'RIGHT': 'receive 15€ with 60% probability or 10€ with 40% probability.'
You have chosen left in that particular row. Therefore, you either get 20€ or

5€, but the probability to get 20€ is higher.

Every screen and every row on each screen has an equal chance to be chosen by the computer to be relevant for your payoff. Considering that every decision you make matters, we advise you to think carefully about each decision you make.

After you made these twelve decisions, ‘Part 2’ will start. Instructions for ‘Part 2’ will be given on the screens themselves. One situation in ‘Part 2’ will be randomly selected to affect your payment. ‘Part 1’ and ‘Part 2’ are completely independent of each other.

Your final payoff will then be the sum of your payoffs from ‘Part 1’ and ‘Part 2’.

If you have no questions, please click ‘OK’ to answer a couple of control questions. These questions will make sure that you have understood the setup. You cannot commence with the experiment unless you answer the control questions correctly.

The experiment will start afterwards!

If you have questions, please raise your hand at any time and an experimenter will provide assistance.

Auction Instructions

You will now participate in an auction against a computer opponent over a good that has a value of 20€. You can bid any amount from 0€ to 20€, and you can specify your bid down to the exact cent.

The computer will bid a random number from 0€ to 20€, down to the exact cent, and each number has an equal probability to be chosen.

If your bid is higher than the computer’s, you will get 20€ minus your bid as your payoff. If your bid is lower than the computer’s, you will get 0€.

If your bids are tied, the winner of the auction is selected randomly and you will receive the payoff of 20€ minus your bid with 50% probability.

Example 1: If you bid 12.41€ and the computer bids 16.53€, your payoff is 0€ as the computer’s bid is higher than yours.
Example 2: If you bid 18.8€ and the computer bids 0.17€, your payoff is 1.2€ (=20-18.8) from this auction, as your bid is higher than the computer’s.

Remember, you can bid any amount from 0€ to 20€. If you win the auction, your payoff is 20€ minus your bid.

Now, please type in how much you want to bid for the good.

Investment Game Instructions

You will now have the opportunity to invest an endowment of 10.00€.

There are two assets you can invest in: STOCKS and BONDS.

The amount you invest in bonds does not give returns. You will get the amount you invested as your payoff for sure.

STOCKS: STOCKS can have higher gains than BONDS, but are more risky. The amount you invest in STOCKS has a 50% chance to be multiplied by 1.5, and a 50% chance to be lost.

You can freely allocate your endowment of 10.00€ between the two assets, down to the exact cent.

Example 1: You invest 10€ in BONDS and 0€ in STOCKS. Your payoff will be 10€.
Example 2: You choose to invest 2.58€ in BONDS and 7.42€ in STOCKS. Your payoff will either be 13.71€ (=7.42*1.5+2.58) with 50% probability, or 2.58€ with 50% probability.
Example 3: You choose to invest all 10€ into STOCKS. Your payoff will either be 15€ (=10*1.5) with 50% probability, or 0€ with 50% probability.

Remember:
You will receive the amount you invest in BONDS as your payoff.

You will receive the amount you invest in STOCKS times 1.5 with 50% probability, and 0€ with 50% probability.

Please choose how much you want to invest in STOCKS (the rest of your endowment will be invested in BONDS):

Text of Control Questions

Risk Preference Elicitation

1.: Suppose that your earnings depend on the following lottery. The computer randomly chooses row '3' to be relevant for your payoff. Your choice was to switch from 'LEFT' to 'RIGHT' at row '7'. How high is your payoff in the best case?
2.: Suppose that your earnings depend on the following lottery. What is the

probability in % for you getting 4.50€ if the computer randomly chooses row '8' to be relevant for your payoff and you clicked radio button '5'?
3.: Suppose that your earnings depend on the following lottery. You have chosen to switch from 'LEFT' to 'RIGHT' at row '2'. The computer randomly chose row '2' to be relevant for your payoff. What is the probability in % that you will get 8.60€?

Benchmark Games

4.: If you invest 7€ in STOCKS, what is the probability in % that you will receive 20.5€?
5.: How much do you receive as payoff if you decided to invest 4€ in STOCKS and you prove to be lucky with your investment?
6.: If you invest 9€ in STOCKS, what is the probability in % that you will receive 22.5€?
7.: Your bid is 13.5€ and the computer's bid is 12.0€. How much is your payoff?
8.: Your bid is 8.0€ and the computer's bid is 13.4€. How much is your payoff?

Risk Preference Elicitation Methods

In this section we report the parameters for the gambles as they were presented to subjects. Note that all the preference elicitation methods described here are represented top-down for simplicity reasons. An example of a bottom-up representation of a particular method is provided afterwards.

Table 13: *SGp method*

Left Option				Right Option			
p_1^L	π_1^L	p_2^L	π_2^L	p_1^R	π_1^R	p_2^R	π_2^R
1	8	-	-	0.62	4	0.38	12
1	8	-	-	0.564	4	0.4364	12
1	8	-	-	0.52	4	0.48	12
1	8	-	-	0.481	4	0.5192	12
1	8	-	-	0.447	4	0.553	12
1	8	-	-	0.411	4	0.589	12
1	8	-	-	0.374	4	0.626	12
1	8	-	-	0.323	4	0.677	12

Table 14: *SGhigh method*

Left Option				Right Option			
p_1^L	π_1^L	p_2^L	π_2^L	p_1^R	π_1^R	p_2^R	π_2^R
1	8	-	-	0.5	4	0.5	10.63
1	8	-	-	0.5	4	0.5	11.16
1	8	-	-	0.5	4	0.5	11.7
1	8	-	-	0.5	4	0.5	12.32
1	8	-	-	0.5	4	0.5	13.04
1	8	-	-	0.5	4	0.5	14.07
1	8	-	-	0.5	4	0.5	15.75
1	8	-	-	0.5	4	0.5	20.31

Table 15: *SGlow method*

Left Option				Right Option			
p_1^L	π_1^L	p_2^L	π_2^L	p_1^R	π_1^R	p_2^R	π_2^R
1	9.5	-	-	0.5	6.1	0.5	12
1	9.5	-	-	0.5	6.61	0.5	12
1	9.5	-	-	0.5	6.89	0.5	12
1	9.5	-	-	0.5	7.09	0.5	12
1	9.5	-	-	0.5	7.24	0.5	12
1	9.5	-	-	0.5	7.38	0.5	12
1	9.5	-	-	0.5	7.51	0.5	12
1	9.5	-	-	0.5	7.66	0.5	12

Table 16: *SGsure method*

Left Option				Right Option			
p_1^L	π_1^L	p_2^L	π_2^L	p_1^R	π_1^R	p_2^R	π_2^R
1	8.91	-	-	0.5	4	0.5	12
1	8.5	-	-	0.5	4	0.5	12
1	8.16	-	-	0.5	4	0.5	12
1	7.85	-	-	0.5	4	0.5	12
1	7.57	-	-	0.5	4	0.5	12
1	7.27	-	-	0.5	4	0.5	12
1	6.96	-	-	0.5	4	0.5	12
1	6.56	-	-	0.5	4	0.5	12

Table 17: *SGall method*

Left Option				Right Option			
p_1^L	π_1^L	p_2^L	π_2^L	p_1^R	π_1^R	p_2^R	π_2^R
0.5	8	0.5	8	0.5	10	0.5	5.34
0.5	10	0.5	5.34	0.5	11	0.5	3.83
0.5	11	0.5	3.83	0.5	12	0.5	2.61
0.5	12	0.5	2.61	0.5	13	0.5	1.83
0.5	13	0.5	1.83	0.5	14	0.5	1.41
0.5	14	0.5	1.41	0.5	15	0.5	1.21
0.5	15	0.5	1.21	0.5	16.5	0.5	1.09
0.5	16.5	0.5	1.09	0.5	20.5	0.5	1.01

Table 18: *PGp method*

Left Option				Right Option			
p_1^L	π_1^L	p_2^L	π_2^L	p_1^R	π_1^R	p_2^R	π_2^R
0.2	9	0.8	7.2	0.2	17.2	0.8	0.45
0.3	9	0.7	7.2	0.3	17.2	0.7	0.45
0.4	9	0.6	7.2	0.4	17.2	0.6	0.45
0.5	9	0.5	7.2	0.5	17.2	0.5	0.45
0.6	9	0.4	7.2	0.6	17.2	0.4	0.45
0.7	9	0.3	7.2	0.7	17.2	0.3	0.45
0.8	9	0.2	7.2	0.8	17.2	0.2	0.45
0.9	9	0.1	7.2	0.9	17.2	0.1	0.45

Table 19: *PGhigh method*

Left Option				Right Option			
p_1^L	π_1^L	p_2^L	π_2^L	p_1^R	π_1^R	p_2^R	π_2^R
0.5	9	0.5	7.2	0.5	10.96	0.5	3.7
0.5	9	0.5	7.2	0.5	11.55	0.5	3.7
0.5	9	0.5	7.2	0.5	12.15	0.5	3.7
0.5	9	0.5	7.2	0.5	12.87	0.5	3.7
0.5	9	0.5	7.2	0.5	13.75	0.5	3.7
0.5	9	0.5	7.2	0.5	15.01	0.5	3.7
0.5	9	0.5	7.2	0.5	17.21	0.5	3.7
0.5	9	0.5	7.2	0.5	23.83	0.5	3.7

Table 20: *PGlow method*

Left Option				Right Option			
p_1^L	π_1^L	p_2^L	π_2^L	p_1^R	π_1^R	p_2^R	π_2^R
0.5	16.09	0.5	7	0.5	3.7	0.5	17.2
0.5	15.3	0.5	7	0.5	3.7	0.5	17.2
0.5	14.41	0.5	7	0.5	3.7	0.5	17.2
0.5	13.35	0.5	7	0.5	3.7	0.5	17.2
0.5	12.18	0.5	7	0.5	3.7	0.5	17.2
0.5	10.85	0.5	7	0.5	3.7	0.5	17.2
0.5	9.29	0.5	7	0.5	3.7	0.5	17.2
0.5	7.35	0.5	7	0.5	3.7	0.5	17.2

Table 21: *PGall method*

Left Option				Right Option			
p_1^L	π_1^L	p_2^L	π_2^L	p_1^R	π_1^R	p_2^R	π_2^R
0.99	7.55	0.01	0	0.81	8.08	0.19	0
0.94	7.93	0.06	0	0.78	8.73	0.22	0
0.89	8.28	0.11	0	0.75	9.28	0.25	0
0.84	8.60	0.16	0	0.72	9.83	0.28	0
0.79	8.98	0.21	0	0.69	10.53	0.31	0
0.74	9.33	0.26	0	0.66	11.33	0.34	0
0.69	9.70	0.31	0	0.63	12.90	0.37	0
0.64	10.05	0.36	0	0.62	28.95	0.38	0

Example of bottom-up representation

Table 22: *PGhigh method; Bottom-Up Appearance*

Left Option				Right Option			
p_1^L	π_1^L	p_2^L	π_2^L	p_1^R	π_1^R	p_2^R	π_2^R
0.5	23.83	0.5	3.7	0.5	9	0.5	7.2
0.5	17.21	0.5	3.7	0.5	9	0.5	7.2
0.5	15.01	0.5	3.7	0.5	9	0.5	7.2
0.5	13.75	0.5	3.7	0.5	9	0.5	7.2
0.5	12.87	0.5	3.7	0.5	9	0.5	7.2
0.5	12.15	0.5	3.7	0.5	9	0.5	7.2
0.5	11.55	0.5	3.7	0.5	9	0.5	7.2
0.5	10.96	0.5	3.7	0.5	9	0.5	7.2

Robustness Checks on Functional Form

In this section we report the regressions from Table 7 with the different assumption that a subject's utility function does not follow CRRA but either PT, CARA, DRRA, IRRA, IARA or DARA. For a discussion of these different assumptions, see the end of Section 3.4.1 and Section 1.1.
The results remain unchanged or provide an even more favorable picture if we make these assumptions as far as significance is concerned. PGhigh becomes a significant explanatory factor under DRRA, and several other methods lose their significance under the other specifications. We conclude that our results are robust to the assumption of the underlying utility function.

Table 23: *Explanatory power - CARA*

	SGp	SGhigh	SGlow	SGsure	SGall	PGp	PGhigh	PGlow	PGall
	**OLS coefficients (with controls)**								
Auction	5.17	4.76	5.47	0.2	-1.82	0.26	-0.04	5.01	-2.2
	(.05)	(.04)	(.04)	(.02)	(.02)	(.02)	(.02)	(.04)	(.03)
Inv. Low	-0.81	-7.65*	-4.57	0.5	-4.51	-2.65	-12.11**	-1.83	-1.72
	(.00)	(.03)	(.00)	(.00)	(.01)	(.00)	(.07)	(.00)	(.00)
Inv. High	-6.87**	-5.48	-2.63	1.82	3.79	-2.93	-5.55	-5.61	0.87
	(.16)	(.12)	(.09)	(.09)	(.11)	(.10)	(.10)	(.12)	(.09)
	**Spearman rank correlation coefficients**								
Auction	.23*	.09	.14	.17	.07	.11	.06	.16	-.13
Inv. Low	.02	.19	.17	.06	.06	.11	.36***	.12	.04
Inv. High	.28**	.28**	.05	0	.03	.13	.26**	.23*	.09

*Notes: Compared to Table 7 in the main text, significance remains the same, and R^2 values are lower. Spearman rank correlation coefficients are unchanged due to the nature of the functional form. Stars are given as follows: *: p<0.05; **: p<0.01; ***: p<0.001*

Table 24: *Explanatory power - prospect theory*

	SGp	SGhigh	SGlow	SGsure	SGall	PGp	PGhigh	PGlow	PGall
	**OLS coefficients (with controls)**								
Auction	-0.68	-0.53	-0.58	0.02	0.29	-0.03	0	-0.59	0.52
	(.05)	(.03)	(.04)	(.02)	(.02)	(.02)	(.02)	(.04)	(.03)
Inv. Low	0.09	0.94*	0.48	-0.01	0.66	0.39	1.48**	0.23	0.44
	(.00)	(.03)	(.00)	(.00)	(.00)	(.00)	(.08)	(.00)	(.00)
Inv. High	0.91**	0.68	0.28	-0.21	-0.58	0.46	0.66	0.65	-0.2
	(.16)	(.12)	(.09)	(.09)	(.11)	(.09)	(.11)	(.12)	(.08)
	**Spearman rank correlation coefficients**								
Auction	.23*	.09	.14	.17	.07	.11	.06	.16	-.13
Inv. Low	.02	.19	.17	.06	.06	.11	.36***	.12	.04
Inv. High	.28**	.28**	.05	0	.03	.13	.26**	.23*	.09

*Notes: Spearman rank correlations and adjusted R^2 values are unchanged compared to Table 7 in the main text due to the similarity of the functional form to CRRA; expected signs are the opposite as for the other funcional forms; stars are given as follows: *: p<0.05; **: p<0.01; ***: p<0.001*

Table 25: *Explanatory power - DRRA*

	SGp	SGhigh	SGlow	SGsure	SGall	PGp	PGhigh	PGlow	PGall
	**OLS coefficients (with controls)**								
Auction	0.56	0.45	0.48	-0.07	-0.2	0.01	-0.03	0.42	-0.4
	(.05)	(.03)	(.04)	(.02)	(.02)	(.02)	(.02)	(.04)	(.04)
Inv. Low	-0.03	-0.76*	-0.38	-0.02	-0.53	-0.32	-1.26***	-0.16	-0.26
	(.00)	(.03)	(.00)	(.00)	(.01)	(.00)	(.09)	(.00)	(.00)
Inv. High	-0.72**	-0.58	-0.24	0.21	0.46	-0.35	-0.55*	-0.53*	0.13
	(.17)	(.12)	(.09)	(.09)	(.11)	(.09)	(.11)	(.12)	(.08)
	**Spearman rank correlation coefficients**								
Auction	.24*	.11	.17	.15	.06	.12	.07	.18	-.1
Inv. Low	.02	.19	.17	.06	.06	.11	.37***	.13	.02
Inv. High	.28**	.29*	.08	.03	.03	.15	.28**	.27**	.1

*Notes: Coefficients, p-values and R^2 values are slightly better compared to Table 7 in the main text; stars are given as follows: *: p<0.05; **: p<0.01; ***: p<0.001*

Table 26: *Explanatory power - IRRA*

	SGp	SGhigh	SGlow	SGsure	SGall	PGp	PGhigh	PGlow	PGall
	**OLS coefficients (with controls)**								
Auction	0.32	0.22	0.24	0.01	-0.11	0.02	0	0.25	-0.27
	(.05)	(.03)	(.04)	(.02)	(.02)	(.02)	(.02)	(.04)	(.04)
Inv. Low	-0.06	-0.42*	-0.19	-0.02	-0.32	-0.18	-0.67***	-0.09	-0.16
	(.00)	(.03)	(.00)	(.00)	(.01)	(.00)	(.10)	(.00)	(.00)
Inv. High	-0.42**	-0.32	-0.13	0.12	0.27	-0.21	-0.29	-0.31*	0.06
	(.17)	(.12)	(.09)	(.09)	(.11)	(.10)	(.11)	(.13)	(.08)
	**Spearman rank correlation coefficients**								
Auction	.23*	.11	.16	.16	.06	.12	.07	.18	-.1
Inv. Low	.02	.2	.18	.06	.06	.11	.37***	.14	.03
Inv. High	.3**	.29**	.08	.03	.03	.15	.28**	.27**	.1

*Notes: Coefficients, p-values and R^2 values are slightly better compared to Table 7 in the main text; stars are given as follows: *: p<0.05; **: p<0.01; ***: p<0.001*

Table 27: *Explanatory power - DARA*

	SGp	SGhigh	SGlow	SGsure	SGall	PGp	PGhigh	PGlow	PGall
	**OLS coefficients (with controls)**								
Auction	3.51	3.23	3.67	-0.16	-1.16	0.84	-0.14	3.32	-1.93
	(.05)	(.04)	(.04)	(.02)	(.02)	(.02)	(.02)	(.04)	(.03)
Inv. Low	-0.43	-5.12**	-2.98	-0.07	-3.07	-1.82	-8.27***	-1.28	-1.73
	(.00)	(.04)	(.00)	(.00)	(.01)	(.00)	(.08)	(.00)	(.00)
Inv. High	-4.59**	-3.68	-1.82	1.12	2.38	-1.76	-3.76	-3.89*	0.27
	(.17)	(.12)	(.09)	(.09)	(.10)	(.09)	(.11)	(.13)	(.08)
	**Spearman rank correlation coefficients**								
Auction	.25*	.11	.18	.15	.07	.12	.07	.18	-.12
Inv. Low	0	.22*	.18	.06	.07	.11	.37***	.12	.06
Inv. High	.28**	.31**	.02	.09	.05	.13	.29**	.27**	.11

*Notes: stars are given as follows: *: p<0.05; **: p<0.01; ***: p<0.001*

Table 28: *Explanatory power - IARA*

	SGp	SGhigh	SGlow	SGsure	SGall	PGp	PGhigh	PGlow	PGall
	**OLS coefficients (with controls)**								
Auction	7.4	6.79	8	0.21	-3.01	0.45	0.16	7.37	-2.94
	(.04)	(.03)	(.04)	(.02)	(.02)	(.02)	(.02)	(.04)	(.03)
Inv. Low	-1.47	-11.2*	-6.84	0.52	-6.48	-3.74	-17.29**	-2.49	-2.19
	(.00)	(.03)	(.00)	(.00)	(.00)	(.00)	(.06)	(.00)	(.00)
Inv. High	-10.09**	-8	-3.62	2.72	5.92	-4.29	-8.02	-7.85	1.43
	(.15)	(.11)	(.09)	(.09)	(.11)	(.10)	(.10)	(.11)	(.09)
	**Spearman rank correlation coefficients**								
Auction	.21*	.18	.11	.17	.07	.11	.03	.13	-.13
Inv. Low	.03	.16	.15	.05	.05	.1	.32***	.13	.03
Inv. High	.28**	.24*	.01	.01	.01	.12	.19	.19	.07

*Notes: Generally lower R^2 than DARA; stars are given as follows: *: p<0.05; **: p<0.01; ***: p<0.001*

Probability Weighting

In this section we report regressions that take probability weighting (PW) into account. For a discussion of these different assumptions, see the end of Section 3.4.1 and Section 1.1. Note that the only methods that change compared to the tables in the above section are SGp, PGp and PGall, as these have changing probabilities over different rows.
The conclusions we drew for Table 7 in the main text remain qualitatively the same if we assume probability weighting.

Table 29: *Explanatory power - CARA+ PW*

	SGp	SGhigh	SGlow	SGsure	SGall	PGp	PGhigh	PGlow	PGall
	**OLS coefficients (with controls)**								
Auction	8.44	4.76	5.47	0.2	-1.82	0.45	-0.04	5.01	-1.83
	(.05)	(.04)	(.04)	(.02)	(.02)	(.02)	(.02)	(.04)	(.03)
Inv. Low	-1.3	-7.65*	-4.57	0.5	-4.51	-4.36	-12.11**	-1.83	-1.9
	(.00)	(.03)	(.00)	(.00)	(.01)	(.00)	(.07)	(.00)	(.00)
Inv. High	-11.22**	-5.48	-2.63	1.82	3.79	-4.82	-5.55	-5.61	0.47
	(.16)	(.12)	(.09)	(.09)	(.11)	(.10)	(.10)	(.12)	(.08)
	**Spearman rank correlation coefficients**								
Auction	.23*	.09	.14	.17	.07	.11	.06	.16	-.13
Inv. Low	.02	.19	.17	.06	.06	.11	.36***	.12	.04
Inv. High	.28**	.28**	.05	0	.03	.13	.26**	.23*	.09

*Notes: Stars are given as follows: *: p<0.05; **: p<0.01; ***: p<0.001*

Table 30: *Explanatory power - PT+ PW*

	SGp	SGhigh	SGlow	SGsure	SGall	PGp	PGhigh	PGlow	PGall
	**OLS coefficients**								
Auction	-1.56	-0.53	-0.58	0.02	0.29	-0.05	0	-0.59	0.09
	(.05)	(.03)	(.04)	(.02)	(.02)	(.02)	(.02)	(.04)	(.02)
Inv. Low	0.19	0.94*	0.48	-0.01	0.66	0.66	1.48**	0.23	0.18
	(.00)	(.03)	(.00)	(.00)	(.00)	(.00)	(.08)	(.00)	(.00)
Inv. High	2.06**	0.68	0.28	-0.21	-0.58	0.76	0.66	0.65	-0.11
	(.16)	(.12)	(.09)	(.09)	(.11)	(.09)	(.11)	(.12)	(.08)
	**Spearman rank correlation coefficients**								
Auction	.23*	.09	.14	.17	.07	.11	.06	.16	-.13
Inv. Low	.02	.19	.17	.06	.06	.11	.36***	.12	.04
Inv. High	.28**	.28**	.05	.00	.03	.29	.26**	.23*	.09

*Notes: Expected signs are the opposite as for the other functional forms; stars are given as follows: *: p<0.05; **: p<0.01; ***: p<0.001*

Table 31: *Explanatory power - DRRA+ PW*

	SGp	SGhigh	SGlow	SGsure	SGall	PGp	PGhigh	PGlow	PGall
	**OLS coefficients (with controls)**								
Auction	1	0.45	0.48	-0.07	-0.2	0.02	-0.03	0.42	-0.32
	(.04)	(.03)	(.04)	(.02)	(.02)	(.02)	(.02)	(.04)	(.03)
Inv. Low	-0.12	-0.76*	-0.38	-0.02	-0.53	-0.53	-1.26***	-0.16	-0.28
	(.00)	(.03)	(.00)	(.00)	(.01)	(.00)	(.09)	(.00)	(.00)
Inv. High	-1.31**	-0.58	-0.24	0.21	0.46	-0.56	-0.55*	-0.53*	0.09
	(.15)	(.12)	(.09)	(.09)	(.11)	(.09)	(.11)	(.12)	(.08)
	**Spearman rank correlation coefficients**								
Auction	.22*	.11	.17	.15	.06	.11	.07	.18	-.1
Inv. Low	.03	.19	.17	.06	.06	.09	.37***	.13	.03
Inv. High	.26*	.29*	.08	.03	.03	.12	.28**	.27**	.11

*Notes: Stars are given as follows: *: p<0.05; **: p<0.01; ***: p<0.001*

Table 32: *Explanatory power - IRRA+ PW*

	SGp	SGhigh	SGlow	SGsure	SGall	PGp	PGhigh	PGlow	PGall
	**OLS coefficients (with controls)**								
Auction	0.76	0.22	0.24	0.01	-0.11	0.01	0	0.25	-0.23
	(.03)	(.03)	(.04)	(.02)	(.02)	(.02)	(.02)	(.04)	(.04)
Inv. Low	-0.63	-0.42*	-0.19	-0.02	-0.32	-0.27	-0.67***	-0.09	-0.16
	(.00)	(.03)	(.00)	(.00)	(.01)	(.00)	(.10)	(.00)	(.00)
Inv. High	-1	-0.32	-0.13	0.12	0.27	-0.28	-0.29	-0.31*	0.03
	(.11)	(.12)	(.09)	(.09)	(.11)	(.09)	(.11)	(.13)	(.08)
	**Spearman rank correlation coefficients**								
Auction	.21*	.11	.16	.16	.06	.11	.07	.18	-.1
Inv. Low	.01	.2	.18	.06	.06	.09	.37***	.14	.04
Inv. High	.27**	.29**	.08	.03	.03	.12	.28**	.27**	.11

*Notes: Stars are given as follows: *: p<0.05; **: p<0.01; ***: p<0.001*

Table 33: *Explanatory power - DARA+ PW*

	SGp	SGhigh	SGlow	SGsure	SGall	PGp	PGhigh	PGlow	PGall
	**OLS coefficients (with controls)**								
Auction	5.83	3.23	3.67	-0.16	-1.16	0.27	-0.14	3.32	-1.53
	(.05)	(.04)	(.04)	(.02)	(.02)	(.02)	(.02)	(.04)	(.03)
Inv. Low	-1.1	-5.12**	-2.98	-0.07	-3.07	-3	-8.27***	-1.28	-1.8
	(.00)	(.04)	(.00)	(.00)	(.01)	(.00)	(.08)	(.00)	(.00)
Inv. High	-8.1**	-3.68	-1.82	1.12	2.38	-3.22	-3.76	-3.89*	-.01
	(.17)	(.12)	(.09)	(.09)	(.10)	(.1)	(.11)	(.13)	(.08)
	**Spearman rank correlation coefficients**								
Auction	.23*	.11	.18	.15	.07	.12	.07	.18	-.12
Inv. Low	0.2	.22*	.18	.06	.07	.11	.37***	.12	.07
Inv. High	.3**	.31**	.02	.09	.05	.15	.29**	.27**	.11

*Notes: Stars are given as follows: *: p<0.05; **: p<0.01; ***: p<0.001*

Table 34: *Explanatory power - IARA+ PW*

	SGp	SGhigh	SGlow	SGsure	SGall	PGp	PGhigh	PGlow	PGall
	**OLS coefficients (with controls)**								
Auction	12.16	6.79	8	0.21	-3.01	0.7	0.16	7.37	-2.03
	(.04)	(.03)	(.04)	(.02)	(.02)	(.02)	(.02)	(.04)	(.03)
Inv. Low	-2.22	-11.2*	-6.84	0.52	-6.48	-6	-17.29**	-2.49	-1.94
	(.00)	(.03)	(.00)	(.00)	(.00)	(.00)	(.06)	(.00)	(.00)
Inv. High	-16.35**	-8	-3.62	2.72	5.92	-7	-8.02	-7.85	1.09
	(.15)	(.11)	(.09)	(.09)	(.11)	(.10)	(.10)	(.11)	(.09)
	**Spearman rank correlation coefficients**								
Auction	.22*	.18	.11	.17	.07	.11	.03	.13	-.14
Inv. Low	.02	.16	.15	.05	.05	.1	.32***	.13	.03
Inv. High	.27**	.24*	.01	.01	.01	.11	.19	.19	.07

*Notes: Stars are given as follows: *: p<0.05; **: p<0.01; ***: p<0.001*

Sample Screenshots

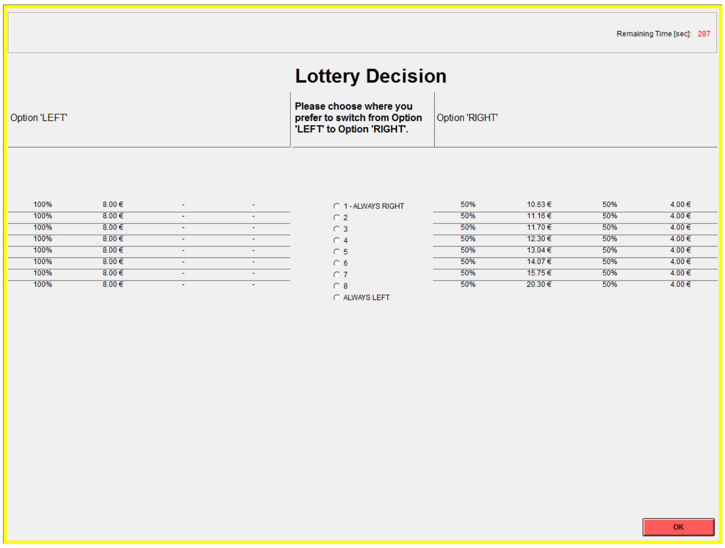

Fig. 5 *Decision-making screen for lotteries; subjects indicated in which row they wanted to switch from the left to the right option by clicking one of the radio buttons in the middle*

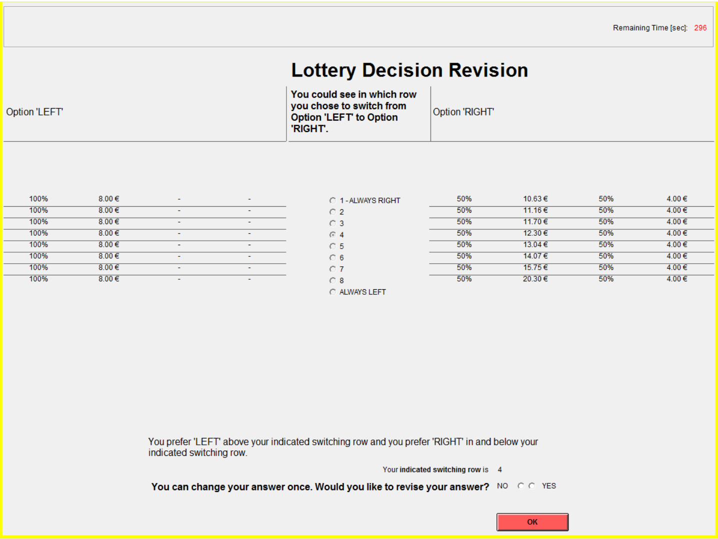

Fig. 6 *Revision screen for lotteries; subjects indicated whether they wanted to revise their first decision*
